# Out of the shadows: Chronic pain in Canadian Armed Forces veterans — Proceedings of a workshop at the 2019 Forum of the Canadian Institute for Military and Veteran Health Research

**DOI:** 10.1080/24740527.2020.1796479

**Published:** 2020-09-10

**Authors:** James M. Thompson, Alexandra Heber, Ramesh Zacharias, Markus Besemann, Gaurav Gupta, Eleni Hapidou, Norm Buckley, Daniel Lamoureux, Kimberly Begley

**Affiliations:** a Department of Public Health Sciences, Queens University, Kingston, Ontario, Canada; b Canadian Institute for Military and Veteran Health Research, Kingston, Ontario, Canada; c Veterans Affairs Canada, Ottawa, Ontario, Canada; d Department of Psychiatry, University of Ottawa, Ottawa, Ontario, Canada; e Department of Anesthesia, Hamilton Health Sciences, McMaster University, Hamilton, Ontario, Canada; f Michael G. DeGroote Pain Clinic, Hamilton, Ontario, Canada; g Canadian Forces Health Services Group Headquarters, Ottawa, Ontario, Canada; h Allan Edwards Pain Management Unit, McGill University, Montreal, Ontario, Canada; i Department of Psychology, Neuroscience and Behaviour, McMaster University, Hamilton, Ontario, Canada; j Department of Psychiatry and Behavioural Neurosciences, McMaster University, Hamilton, Ontario, Canada; k Michael G. DeGroote Institute for Pain Research and Care, Hamilton, Ontario, Canada; l Department of Anesthesia, Michael G. DeGroote School of Medicine, McMaster University, Kingston, Ontario, Canada; m Canadian Armed Forces, Ottawa, Ontario, Canada; n Chronic Pain Network, McMaster University, Hamilton, Ontario, Canada

**Keywords:** chronic pain, pain, veterans, pain clinics, military personnel

## Abstract

This commentary summarizes proceedings of a workshop on chronic pain in military personnel and veterans (released personnel) at the Annual Forum of the Canadian Institute for Military and Veteran Health Research in Gatineau and Ottawa on October 22, 2019. The extent and impact of chronic pain among Canadian Armed Forces (CAF) veterans and their families is significant and has been underappreciated, largely due to limited disclosure by serving and veteran military personnel, stemming from a fear of stigmatization. Living with pain is seen as a fact of life in military cultures, something to be endured and not discussed. Though progress is being made in reducing the stigma of mental illness, the discourse on chronic pain remains censored. This workshop’s goal was to bring the discussion of chronic pain out of the shadows in the search for ways to help veterans and active service personnel living with chronic pain. Many points of view were brought forward at this first national Canadian multidisciplinary gathering of researchers, veterans with lived experience, clinicians, and policymakers. A CAF member described his lived experience with constant chronic pain. Clinicians described aspects of chronic pain in military personnel and veterans whom they treat in their clinics. Dr. Ramesh Zacharias described the new Chronic Pain Center of Excellence for Canadian Veterans that will be established with funding from Veterans Affairs Canada. Dr. Norman Buckley highlighted collaboration with the existing Chronic Pain Network funded by the Canadian Institute for Health Research. Audience members identified a diverse variety of issues.

## RÉSUMÉ

Ce commentaire résume les actes d’un atelier sur la douleur chronique chez le personnel militaire et les anciens combattants (personnel libéré) tenu dans le cadre du Forum annuel de l’Institut canadien de recherche sur la santé des militaires et des vétérans à Gatineau et Ottawa le 22 octobre 2019. L’étendue et l’effet de la douleur chronique chez les anciens combattants des Forces armées canadiennes (FAC) et leurs familles sont importantes et ont été sous-estimées, en grande partie en raison de la divulgation limitée par le personnel militaire en service et les anciens combattants, découlant de la peur de la stigmatisation. Le fait de vivre avec la douleur est considéré comme faisant partie de la vie dans les cultures militaires, quelque chose qu’il faut endurer et dont il ne faut pas discuter. Bien que des progrès aient été réalisés dans la réduction de la stigmatisation de la maladie mentale, le discours sur la douleur chronique continue d’être censuré. L’objectif de cet atelier était de faire sortir de l’ombre la discussion sur la douleur chronique afin de chercher des moyens d’aider les anciens combattants et le personnel de service actif vivant avec la douleur chronique. De nombreux points de vue ont été exprimés lors de cette première rencontre multidisciplinaire nationale canadienne réunissant des chercheurs, des anciens combattants ayant vécu l’expérience de la douleur chronique, des cliniciens et des décideurs. Un membre des FAC a décrit l’expérience de douleur chronique qu’il a vécue. Les cliniciens ont décrit les aspects de la douleur chronique chez le personnel militaire et les anciens combattants qu’ils traitent dans leurs cliniques. Le Dr Ramesh Zacharias a décrit le nouveau Centre d’excellence sur la douleur chronique pour les vétérans canadiens qui sera établi grâce au financement d’Anciens Combattants Canada. Le Dr Norman Buckley a souligné la collaboration avec le Réseau de douleur chronique existant financé par l’Institut canadien de la recherche en santé. Les membres de l’audience ont relevé divers problèmes.

## Introduction

This commentary summarizes the proceedings of a workshop on chronic pain in military personnel and veterans (released personnel) held at the annual Forum of the Canadian Institute for Military and Veteran Health Research in Gatineau and Ottawa on October 22, 2019. The extent and implications of chronic pain in the lives of Canadian Armed Forces (CAF) veterans and their families are significant.^1,2^ Though progress is being made in reducing the stigma of mental illness, censorship of chronic pain discourse remains entrenched. Anecdotally, chronic pain has tended to be underappreciated in military populations due in large part to stigma.^3^ Living with pain is seen as a fact of life in military cultures, something to be endured and not discussed.^3,4^ This workshop was the first national Canadian multidisciplinary gathering of researchers, veterans and serving military with lived experience, clinicians, and policymakers convened to address chronic pain in veterans. The goal was to bring chronic pain out of the shadows to help veterans living with chronic pain.

Recent findings from the groundbreaking Life After Service Studies (LASS) conducted by Statistics Canada in 2010, 2013, and 2016 in collaboration with scientists at Veterans Affairs Canada and the Department of National Defense helped to spotlight the issue. The LASS surveys found that chronic pain is prevalent among CAF veterans released since 1998. In the 2010 survey, 64% answered “yes” to “Do you have any pain or discomfort that is always present?” (41%) or “Do you have any pain or discomfort that reoccurs from time to time?” (23%).^2^ In the 2013 and 2016 surveys, 37% and 41% respectively answered “no” to the Health Utilities Index question “Are you usually free of pain or discomfort?,” about twice the prevalence of the Canadian general population after adjusting for differences in age and sex.^5–7^ The greater prevalence of chronic pain in military veterans is not surprising given the rigors of military service and the higher incidence of potentially predisposing factors such as adverse childhood experiences.^8^

In the LASS surveys of CAF veterans adjusting to postmilitary life, 59% of those who reported difficult adjustment to civilian life had chronic pain.^8^ About half (54%) with chronic pain also reported having pain-related interference with work in the prior month.^7^ More than three quarters (77%) of those with chronic pain had some degree of life stress on most days. Chronic pain usually begins in the context of a physical injury or illness but is highly correlated with the presence of mental health conditions. Of those with the types of self-reported diagnosed chronic physical health conditions asked about in the surveys,^9^ 62% had chronic pain or discomfort, and 63% of those with chronic pain had mental health conditions. Chronic physical health conditions that often are associated with chronic pain such as musculoskeletal disorders, gastrointestinal disorders, diabetes, and migraine were more common in CAF veterans released from 1998 onwards than in the Canadian general population and were statistically associated with suicidal ideation.^9,10^

## A Life Lived with Constant Chronic Pain

Master Warrant Officer Daniel Lamoureux spoke about his lived experience of constant chronic pain. A long-serving CAF member specializing in the arduous search and rescue trade, he has made over a thousand parachute jumps and over 300 SCUBA dives in the context of physically and mentally challenging training and rescues. MWO Lamoureux described how he has been living with chronic pain for 15 to 20 years. He began by saying that it had been hard for him to talk to others about his pain, because it is not something that military personnel like to talk about. He explained the impacts of chronic pain on his work and personal life and the mental health challenges that he experiences. Pain is always present and takes over his life, constantly challenging his acceptance of the pain. MWO Lamoureux said that he has experienced financial impacts, such as paying for some treatments not covered by his disability benefits. He spoke of the high value of the care that he receives from his CAF health care team. He spoke of the daunting thoughts of making the transitions to a role outside search and rescue and eventually to postmilitary life.

## Clinicians Working with Military Personnel and Veterans

LCol Markus Besemann submitted a presentation on the experience of chronic pain and its treatment in serving military personnel. He pointed to the importance of the polytrauma clinical triad of posttraumatic stress disorder, chronic pain, and concussion/traumatic brain injury. He mentioned the role of adverse childhood experiences, which are more prevalent in CAF personnel than in the general population. Adverse childhood experiences can influence all aspects of adult health, including the development of chronic pain. Despite all reasonable medical efforts, functional disability related to chronic pain remains a significant problem in serving military populations. Often, two patients with similar pain conditions have very different levels of functioning and coping, for a variety of reasons. The World Health Organization’s International Classification of Functioning, Disability and Health framework identifies personal and environmental factors as being important in the genesis of limitations to participation in life roles.^11^ These limitations are critical in the context of the development and management of chronic painful conditions. Dr. Besemann pointed out the importance of understanding how much of one’s pain is inherent in the sensation versus the person’s reaction to the sensation. He spoke about the importance of meaning-making and life stories in rehabilitation for people living with chronic pain. Physical performance is a key element of the military identity, and pain limitations significantly challenge military members’ identities.

Dr. Gaurav Gupta reported that chronic pain and musculoskeletal conditions are the most common cause for medical employment limitations and medical release in CAF serving personnel. He described the mid-20th-century groundbreaking work by physician and former circus strongman Dr. John Bonica. Dr. Bonica originated modern biopsychosocial and interdisciplinary approaches to managing chronic pain. He highlighted evolving research into the role of the gut microbiome, connections between psychology and physiology, and the importance of social connections in understanding and treating chronic pain conditions.

LTG (ret’d) Eric Schoomaker, professor at the U.S. Uniformed Services University of the Health Sciences and former U.S. Army Surgeon General, spoke in his Forum keynote address following the workshop about the intersection of mind and body in chronic pain and the importance of a biopsychosocial approach to chronic pain.^12^ He suggested that we have known for 50 years about the importance of team care and complementary, alternative therapies in treating chronic pain, whereas the evidence for commonly prescribed opioids is much more limited. Dr. Schoomaker emphasized the importance of a comprehensive, whole-person, interdisciplinary, stepped care approach to chronic pain management that aims to restore sufferers to participation in life roles.

Dr. Eleni Hapidou reported on data available for CAF veterans who had attended the Michael G. DeGroote Pain Clinic at Hamilton Health Sciences and McMaster University. She and her student, Jane Jomy, described chronic pain management outcomes in 68 veterans and 68 non-veterans attending the intensive, 4-week chronic pain management program at their pain clinic during the 4-year period June 2015 to August 2019.^13^ Groups were matched for age and gender. They found that veterans more often than non-veterans had greater benefits for measures of catastrophizing, kinesiophobia, sensitivity to pain traumatization, pain acceptance, stages of change, and pain coping. Patients were equally satisfied with the program, but the DeGroote pain clinic case managers’ evaluations showed that veterans more often achieved greater benefit. The two groups were similar except that veterans entered the clinic on average 16.8 years after pain onset versus 5.0 years for non-veterans. Dr. Hapidou and Jane Jomy felt that their evidence will provide insights into how veterans engage differently than non-veterans in the program. They highlighted the effectiveness of their interdisciplinary pain management program, recommending further research into the unique pain experience of veterans.

## Chronic Pain Center of Excellence for Canadian Veterans

In July 2019, in response to the clear need for more knowledge about managing chronic pain in CAF veterans, the Minister of Veterans Affairs Canada announced US$20.1 million funding over 5 years and US$5 million per year ongoing for a Chronic Pain Center of Excellence (CoE) for Canadian veterans at McMaster University.^14^ Dr. Ramesh Zacharias was introduced as the inaugural chief executive officer and medical director for the CoE. He summarized McMaster’s strengths as a home for the CoE and said that they have begun national consultations with veterans and their families, research centers, clinicians, and policymakers through a series of traveling consultations across the country. The CoE intends to focus on four areas: leadership, training, evidence-based care, and research. The objectives are to establish research priorities, drive progress toward ensuring well-being in veterans living with chronic pain, foster the development and implementation of policies and strategies, identify and assess new approaches and best practices, build research capacity, and foster partnerships and networks.

## Chronic Pain Network

Dr. Norm Buckley is scientific director of the Michael G. DeGroote Institute for Pain Research and Care at McMaster University and nominated principal investigator of the Chronic Pain Network (CPN) funded by the Canadian Institutes of Health Research.^15^ He explained that the CPN’s vision is to change the way pain is managed in Canada through increased collaboration among researchers, creation of a structure to bring research findings to policymakers, enhancing health care provider training in pain, and establishing effective partnerships with patients. He described the CPN’s reach and research productivity, noting that the CPN will work collaboratively with the CoE. He pointed out that the government’s establishment of the CoE for veterans is a major step toward improving understanding of and care for chronic pain.

## Audience and Panel Discussion

Discussion between the audience and panel raised a number of diverse points about barriers, gaps, and opportunities. Knowledge translation from research to practice remains a significant challenge. Evidence and evaluation tools exist but are not readily available outside academia. There is value in learning from an Australian education initiative that targets assumptions and beliefs about chronic pain among health care providers and the general public. Prevention could include addressing pain management in military personnel early in their careers to prevent chronic pain, reduce stigma, and prepare them for living with chronic pain. There are opportunities to improve chronic pain management across the military to civilian transition. Barriers to care include financial costs for treatments that are not paid for by the public health system or reimbursed by insurers. There is need for more openness to therapies that have been labeled in the past as “adjuncts.” Chronic pain management remains siloed between health care disciplines. The roles of case managers, navigators, and peer support were emphasized. It is important to connect with primary care providers, given their key roles in assisting patients with chronic pain. Geography is a barrier in Canada for veterans who face long travel times for specialized care. Research funding levels have not reflected the burden of chronic pain, but the recent investments by the federal government are beginning to correct that imbalance.

## The Way Forward

This workshop helped to frame the discussion around chronic pain in Canadian military personnel and veterans. There are substantial new resources for addressing chronic pain in veterans (Table 1), including recent new, ongoing federal government funding for the new Chronic Pain CoE; the Center of Excellence on Post Traumatic Stress Disorder and Related Mental Health Conditions at the Royal Ottawa Mental Health Center; and the Canadian Institute for Military and Veteran Health Research based at Queens University and the Royal Military College of Canada in Kingston.^17,18^ The challenges of preventing, treating, and living with chronic pain are significant, but the glass is now more than half full. New research planned and underway will both enhance understanding of the extent of chronic pain in CAF veterans and expand the evidence base for best practices. Readers interested in learning more about the workshop presentations and the Chronic Pain CoE may contact the authors or visit the CoE website.^21^

**Table 1.** Infrastructure addressing chronic pain in Canadian Veterans

Infrastructure element	Role
Provincial/territorial health care systems	Delivers patient care in rural and urban clinics and hospitals across Canada. Patient care is funded from public and private sources
Multidisciplinary pain centers	Highly specialized, with multiple health care disciplines colocated in academic urban centers. Provides patient care, research, and teaching of health professionals. Funded largely from public sources^16^
Multidisciplinary pain clinics	Specialized, interdisciplinary clinics providing patient care but not necessarily engaged in research and teaching health professionals. Funded from private and some public sources^16^
Chronic Pain Center of Excellence for Canadian veterans, based at McMaster University	Establishes research priorities, drives progress toward ensuring well-being in veterans living with chronic pain, fosters policies and strategies, identifies and assesses new approaches and best practices, builds research capacity, and fosters partnerships and networks. Funded by Veterans Affairs Canada.^14,17^
Chronic Pain Network, based at McMaster University	National collaboration of researchers in multidisciplinary pain centers. Promotes research and knowledge transfer. Funded by a grant from the Canadian Institute for Health Research Strategy for Patient-Oriented Research program^15^
Center of Excellence in Post Traumatic Stress Disorder and Related Mental Health Conditions, based at The Royal Ottawa	Supports knowledge networks with researchers across the country to increase expertise on military and veteran mental health, suicide prevention, and substance use disorder. Funded by Veterans Affairs Canada^18^
Canadian Institute for Military and Veteran Health Research, based at Queen’s University and the Royal Military College of Canada	Enhances the lives of Canadian military personnel, veterans, and their families by harnessing national capacity for research. Provides infrastructure to enable research, enhance knowledge accessibility, and foster collaborations in research and knowledge exchange. Funded by Veterans Affairs Canada and other agencies^19^
Veterans Affairs Canada	Federal government department. Delivers programs to support the well-being of veterans and their families and to commemorate the achievements and sacrifices of Canadians during periods of war, military conflict, and peace^19^
Canadian Armed Forces/Department of National Defense	Unified armed forces of Canada and the federal government department managing and directing the CAF. The CAF provides health, wellness, and spiritual services to support CAF members and their families in service and during transition to postservice life^20^

## Disclosure of Interest

In accordance with Taylor & Francis policy and their obligations to researchers, the authors make the following reports. Dr. Thompson is a paid consultant to the Canadian Institute for Military and Veteran Health Research. Dr. Heber reports no conflicts of interest. Dr. Zacharias is the medical director of the Michael G. DeGroote Pain Clinic at Hamilton Health Science and is the president, chief executive officer, and medical director of the Chronic Pain Center of Excellence for Canadian Veterans. Dr. Besemann reports no conflicts of interest. Dr. Gupta reports no conflicts of interest. Dr. Hapidou reports no conflict of interest. Dr. Buckley is scientific director of the Michael G. DeGroote Institute for Pain Research and Care, nominated principal applicant for the Chronic Pain Network funded by the SPOR program at CIHR, and scientific director of the Chronic Pain Center of Excellence Funded by Veterans Affairs Canada. MWO Lamoureux does not have any potential conflicts of interest. K. Begley is the managing director of the Chronic Pain Network funded by the SPOR program at CIHR and consultant with the Chronic Pain Center of Excellence funded by Veterans Affairs Canada.
